# Recessively Inherited Deficiency of Secreted WFDC2 (HE4) Causes Nasal Polyposis and Bronchiectasis

**DOI:** 10.1164/rccm.202308-1370OC

**Published:** 2024-04-16

**Authors:** Gerard W. Dougherty, Lawrence E. Ostrowski, Tabea Nöthe-Menchen, Johanna Raidt, Andre Schramm, Heike Olbrich, Weining Yin, Patrick R. Sears, Hong Dang, Amanda J. Smith, Achim G. Beule, Rim Hjeij, Niels Rutjes, Eric G. Haarman, Saskia M. Maas, Thomas W. Ferkol, Peadar G. Noone, Kenneth N. Olivier, Diana C. Bracht, Pascal Barbry, Laure-Emmanuelle Zaragosi, Morgane Fierville, Sabine Kliesch, Kai Wohlgemuth, Julia König, Sebastian George, Niki T. Loges, Agathe Ceppe, Matthew R. Markovetz, Hong Luo, Ting Guo, Hoda Rizk, Tarek Eldesoky, Katrin Dahlke, Karsten Boldt, Marius Ueffing, David B. Hill, Yuan-Ping Pang, Michael R. Knowles, Maimoona A. Zariwala, Heymut Omran

**Affiliations:** ^1^ Department of General Pediatrics,; ^2^ Department of Otorhinolaryngology, and; ^3^ Department of Clinical and Surgical Andrology, Centre of Reproductive Medicine and Andrology, University Hospital Muenster, Muenster, Germany;; ^4^ Department of Pediatrics,; ^5^ Marsico Lung Institute/Cystic Fibrosis Research and Treatment Center,; ^6^ Department of Medicine,; ^7^ Department of Physics and Astronomy, and; ^8^ Department of Pathology and Laboratory Medicine, University of North Carolina at Chapel Hill, Chapel Hill, North Carolina;; ^9^ Department of Pediatric Pulmonology and Allergy, Emma Children’s Hospital, Amsterdam, the Netherlands;; ^10^ Department of Human Genetics, Amsterdam Reproduction and Development Research Institute, Amsterdam University Medical Center, University of Amsterdam, Amsterdam, the Netherlands;; ^11^ Université Côte d’Azur, CNRS, Institut Pharmacologie Moléculaire et Cellulaire, Sophia-Antipolis, France;; ^12^ Department of Pulmonary and Critical Care Medicine, the Second Xiangya Hospital, Central South University, Changsha, China;; ^13^ Department of Pediatrics, Faculty of Medicine, University of Mansoura, Mansoura, Egypt;; ^14^ Institute for Ophthalmic Research and Core Facility for Medical Proteomics, Tübingen, Germany;; ^15^ Eberhard Karls University Tübingen, Tübingen, Germany; and; ^16^ Department of Molecular Pharmacology and Experimental Therapeutics, Mayo Clinic, Rochester, Minnesota

**Keywords:** *Pseudomonas*, infertility, chronic airway disease

## Abstract

**Rationale:**

Bronchiectasis is a pathological dilatation of the bronchi in the respiratory airways associated with environmental or genetic causes (e.g., cystic fibrosis, primary ciliary dyskinesia, and primary immunodeficiency disorders), but most cases remain idiopathic.

**Objectives:**

To identify novel genetic defects in unsolved cases of bronchiectasis presenting with severe rhinosinusitis, nasal polyposis, and pulmonary *Pseudomonas aeruginosa* infection.

**Methods:**

DNA was analyzed by next-generation or targeted Sanger sequencing. RNA was analyzed by quantitative PCR and single-cell RNA sequencing. Patient-derived cells, cell cultures, and secretions (mucus, saliva, seminal fluid) were analyzed by Western blotting and immunofluorescence microscopy, and mucociliary activity was measured. Blood serum was analyzed by electrochemiluminescence immunoassay. Protein structure and proteomic analyses were used to assess the impact of a disease-causing founder variant.

**Measurements and Main Results:**

We identified biallelic pathogenic variants in WAP four-disulfide core domain 2 (*WFDC2*) in 11 individuals from 10 unrelated families originating from the United States, Europe, Asia, and Africa. Expression of *WFDC2* was detected predominantly in secretory cells of control airway epithelium and also in submucosal glands. We demonstrate that WFDC2 is below the limit of detection in blood serum and hardly detectable in samples of saliva, seminal fluid, and airway surface liquid from *WFDC2*-deficient individuals. Computer simulations and deglycosylation assays indicate that the disease-causing founder variant p.Cys49Arg structurally hampers glycosylation and, thus, secretion of mature WFDC2.

**Conclusions:**

*WFDC2* dysfunction defines a novel molecular etiology of bronchiectasis characterized by the deficiency of a secreted component of the airways. A commercially available blood test combined with genetic testing allows its diagnosis.

**At a Glance Commentary**

**Scientific Knowledge on the Subject**

Chronic respiratory disorders characterized by bronchiectasis have underlying genetic or environmental causes, but most remain idiopathic. The secreted protein WFDC2, also known as HE4, is a member of the WFDC (WAP four-disulfide core) domain family and implicated in host immune defense. WFDC2 is a well-studied serum biomarker for ovarian cancer and other malignancies and is elevated in serum from subjects with cystic fibrosis and idiopathic pulmonary fibrosis.

**What This Study Adds to the Field**

Here we identify disease-causing variants in *WFDC2* that underlie a unique and severe respiratory disorder characterized by bronchiectasis in all lung fields, chronic rhinosinusitis, and lung infection by *Pseudomonas aeruginosa* resembling the clinical phenotype of cystic fibrosis, primary ciliary dyskinesia, and inborn errors of immunity. This work highlights a novel Mendelian cause of chronic destructive airway disease that results from deficiency of secreted WFDC2. The diagnosis can be suspected based on measurement of serum or saliva WFDC2/HE4 concentrations and confirmed by genetic testing. Because of the relatively small size of WFDC2 and its function in extracellular spaces, replacement therapy may be a potential option. This study adds to our understanding of the causes of bronchiectasis.

Bronchiectasis is a pulmonary disorder defined by persistent, pathologic dilatation of the bronchi associated with chronic cough, sputum production, and recurrent respiratory infections (1). Bronchiectasis exhibits phenotypic variability ranging from local abnormalities to pan-lobar defects and from mild dilatation of bronchi to cystic abnormalities (2). The underlying causes of bronchiectasis are remarkably heterogeneous and may be observed after infection or with recurrent aspirations. Several diseases are commonly associated with bronchiectasis (2, 3), including cystic fibrosis (CF) (3), primary ciliary dyskinesia (PCD) (3, 4), allergic bronchopulmonary aspergillosis (4), and chronic nontuberculous mycobacteria infections (5–7), as well as inborn errors of immunity (IEI) (2–4). Bronchiectasis is sometimes also associated with asthma and chronic obstructive pulmonary disease. However, the majority of bronchiectasis cases remain idiopathic (3, 4), highlighting the need for more research in this area.

The pathological mechanisms underlying bronchiectasis are varied. Evidence suggests that the final common pathway results from a multifactorial combination of chronic or recurrent infection, impaired clearance of pathogens, excessive inflammatory response, and damage, followed by an abnormal remodeling of the lung tissue (2, 5). In up to 80% of patients with bronchiectasis, particular pathogens, including *Pseudomonas aeruginosa*, *Haemophilus influenza*, and nontuberculous mycobacteria, can be cultured from sputum samples (2). Bronchiectasis associated with *P. aeruginosa* infection is accompanied by an increased decline of lung function, exacerbation frequency, hospitalization risk, and mortality (2, 8).

In this study, we performed next-generation sequencing to identify possible genetic defects in unsolved cases of bronchiectasis. We identified biallelic WAP four-disulfide core domain 2 (*WFDC2*) variants in 11 individuals from 10 unrelated families. These individuals presented with symptoms and findings that resembled CF (MIM 219700) and PCD (MIM 244400), including bronchiectasis throughout all lung fields (upper, middle, and lower lobes) and *P. aeruginosa* infection. In addition, severe chronic rhinosinusitis (CRS) with nasal polyposis was a hallmark. WFDC2, also referred to as HE4 (Human Epididymis Protein 4), belongs to the WFDC (WAP four-disulfide core) domain protein family. This study highlights deficiency of WFDC2 as a novel cause of chronic destructive airway disease, with a molecular etiology distinct from other genetic airway diseases such as CF, PCD, and IEI.

## Methods

### Patients and Study Design

Patients were recruited at the University Hospital Muenster and the University of North Carolina, as well as collaborating institutions. We prioritized in-depth genetic analysis and clinical workup of individuals with chronic airway disease characterized by bronchiectasis who were not suspected for CF or PCD because of lack of pathogenic variants detected by *CFTR* and PCD panel genetic testing, respectively. Initially, *WFDC2* variants were identified in exomes of eight individuals from seven unrelated families (>600 unsolved exomes). A targeted *WFDC2* Sanger screening was performed in 1,229 individuals with chronic respiratory symptoms with or without bronchiectasis and identified 2 unrelated individuals (OP-398 II1 and OP-1837 II1), who were further analyzed by next-generation sequencing to assess *CFTR* and PCD-causing variants. This search was broadened to newly available exomes and identified *WFDC2* variants in OP-4474 II1, who presented with nasal polyposis but not bronchiectasis at 7 years of age. Written informed consent was obtained from all individuals and family members in this study according to protocols approved by the Institutional Ethics Review Board of the University Muenster and the Institutional Review Board at the University of North Carolina; studies were performed in compliance with ethical regulations and collaborating institutions. Additional information detailing genetic, molecular, cellular, proteomic, and protein structure analysis is provided in the online supplement.

## Results

### Genetic Analysis

**Figure 1.**
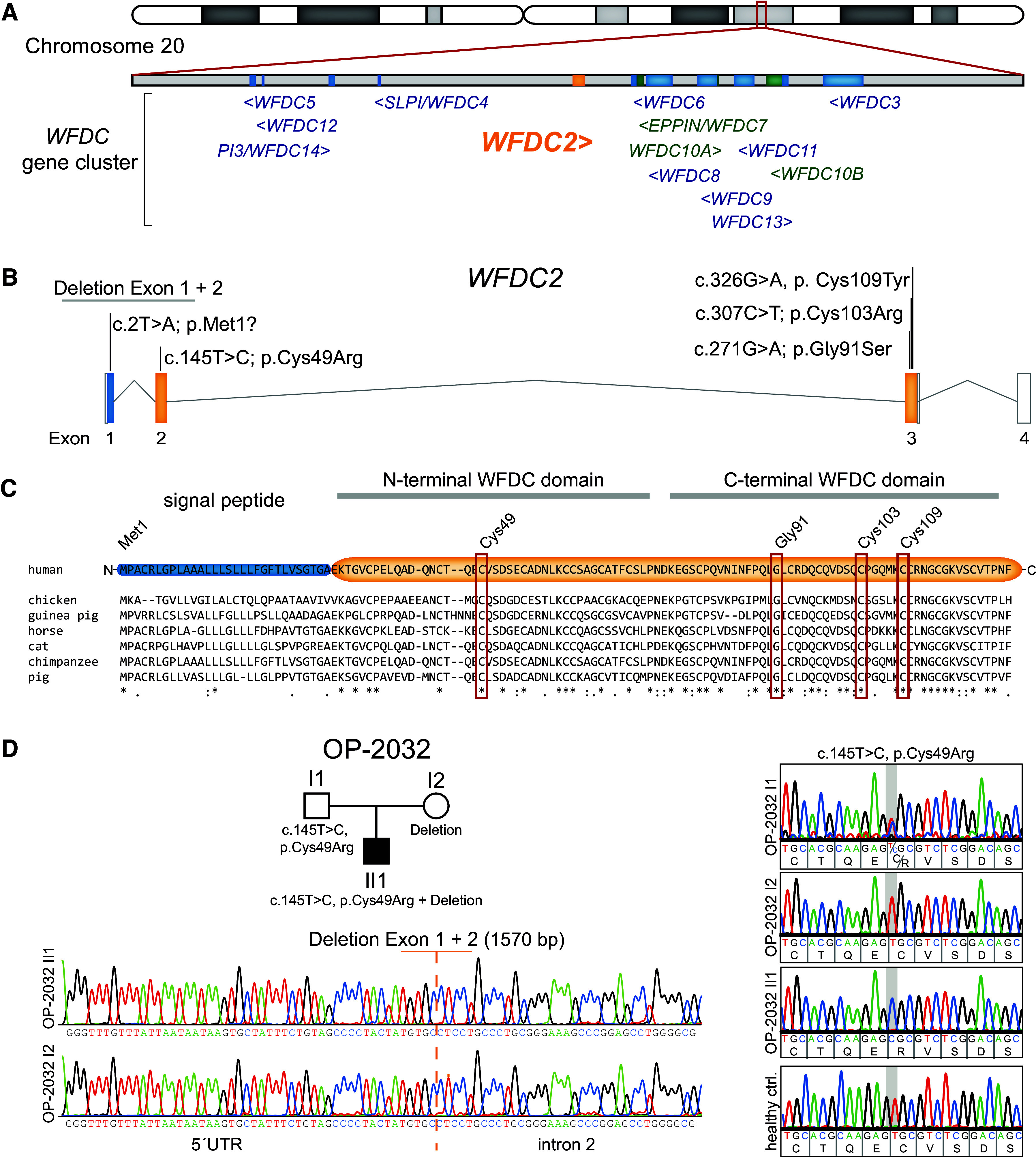
Identification of biallelic *WFDC2* variants in 11 individuals from 10 global families. (*A*) *WFDC2* localizes within the narrow *WFDC* gene cluster on human chromosome 20q13 with 14 of 18 members of the *WFDC* gene family. (*B*) The canonical transcript of *WFDC2* (ENST00000372676) comprises four exons (Figure E3B); disease-causing *WFDC2* variants identified in this study are indicated above respective exons. (*C*) *WFDC2* encodes a secreted protein containing two WFDC domains, which are highly conserved throughout vertebrates as shown by multiple sequence alignments. Exon 1 encodes the signal peptide, which is cleaved before secretion. Exon 2 and exon 3 encode the N- and C-terminal WFDC domains, respectively. The WFDC domains comprise eight cysteine residues, whose disulfide bridges form a characteristic core motif. We identify seven distinct *WFDC2* variants, including a start codon variant, two distinct large deletions, and four missense variants (indicated by red rectangles), affecting highly conserved residues within the WFDC domains. (*D*) OP-2032 II1 harbors *WFDC2* variant c.145C>T, p.Cys49Arg, as well as a 1,570-bp deletion spanning exons 1 and 2. Segregation analysis verifies these variants are inherited from the father and mother, respectively. Additional pedigrees and Sanger sequencing are shown in the data supplement (Figure E1).

Several groups identified ultrarare and pathogenic *WFDC2* variants in individuals with bronchiectasis and connected through GeneMatcher (9) (Table 1). Using whole-exome sequencing (WES), an apparently homozygous start codon variant (c.2T>A; p.Met1?) was identified in *WFDC2* (Figures 1A–1C) in individual UNC-376 II1. Segregation analysis revealed the mother as carrier but the father to be wild type, indicating the possibility of a large deletion on the paternal allele at this locus (*see* Figure E1G in the online supplement). The exact breakpoints for this deletion are unknown; however, heterozygosity for two SNPs encompassing a distance of 137,425 base pairs including the *WFDC2* locus (rs2072786 and rs2143221) were identified; we confirmed parental identity using 21 markers located on six different chromosomes (Table E1).

**Table 1.** Summary of Genetic and Clinical Findings of Individuals with Biallelic *WFDC2* Variants

Individual	Sex	Age (yr)	WFDC2 Variant	Cough	Bronchiectasis	Nasal Polyposis	P.a.	FEV_1_% Predicted	nNO (nl/min)
UNC-376 II1	F	22	c.2T>A; p.Met1? het. deletion, het.	Yes	Yes	Yes	Yes	73	16.8
OP-2147 II1	M	30	c.145T>C; p.Cys49Arg; hom.	Yes	Yes	Yes	Yes	79	5.5
OP-2032 II1	M	14	c.145T>C; p.Cys49Arg; het. deletion exon 1-2; het.	Yes	Yes	Yes	Yes	67	5.3
OP-1837 II1	M	52	c.145T>C; p.Cys49Arg; hom.	N/A	Yes	Yes	Yes	N/A	16.6
OP-4281 II1	M	19	c.145T>C; p.Cys49Arg; hom.	Yes	Yes	Yes	Yes	38	2.3
UNC-231 II1	F	52	c.145T>C; p.Cys49Arg; hom.	Yes	Yes	Yes	Yes	41.6	33.7
OP-398 II1	F	26	c.145T>C; p.Cys49Arg; het. c.271G>A; p.Gly91Ser; het.	N/A	N/A	Yes	N/A	N/A	N/A
UNC-186 II1	M	46*	c.145T>C; p.Cys49Arg; het. c.307T>C; p.Cys103Arg; het.	Yes	Yes	Yes	Yes	64	10.8
UNC-186 II2	F	51*	c.145T>C; p.Cys49Arg; het. c.307T>C; p.Cys103Arg; het.	Yes	Yes	Yes	Yes	25^†^	17.8
CSU-150 II2	F	48*	c.307T>C; p.Cys103Arg; hom.	Yes	Yes	Yes	Yes	22	12
OP-4474 II1	M	7	c.326G>A; p.Cys109Tyr; hom.	Yes	No	Yes	N/A	67	N/A

*Definition of abbreviations*: het = heterozygous; hom = homozygous; N/A = data not available; nNO = nasal nitric oxide production rate; P.a. = *Pseudomonas aeruginosa* infection of the airways.

Individuals UNC-376 II1, OP-2147 II1, OP-2032 II1, OP-398 II1, UNC-186 II1, UNC-186 II2, and OP-4474 II1 reported otitis media. Individuals OP-2147 II1, UNC-231 II1, and UNC-186 II1 reported neonatal respiratory distress syndrome. All 11 individuals reported chronic sinusitis. All 11 individuals had normal situs composition (situs solitus) (*see also* Patient History in the online supplement).

* Deceased.

^†^ FEV_1_ value before lung transplant.

In addition, WES detected a missense variant, c.145T>C, p.Cys49Arg, in *WFDC2* in individual OP-2032 II1 (Figure 1D). This variant alters a highly conserved cysteine residue essential for the functional WFDC domain (10) (Figure 1C). By Sanger sequencing, we confirmed the inheritance of the variant from the father (heterozygous carrier) but detected only the wild-type allele in the mother. A careful reanalysis of the WES data revealed that in OP-2032 II1, the coverage of *WFDC2* exons 1 and 2 was approximately 50%, suggesting a heterozygous deletion comprising these exons. By primer walking and bridging PCR, we subsequently identified the breakpoints that defined a deletion of 1,570 bp spanning *WFDC2* exons 1 and 2 in the heterozygous state in both the affected individual, OP-2032 II1, and the mother, OP-2032 I2 (Figure 1D). This bridging PCR amplified only a wild-type size product in UNC-376 III1, demonstrating the deletion to be distinct from that detected in OP-2032 II1.

Notably, WES identified *WFDC2* variant c.145T>C, p.Cys49Arg in individuals OP-2147 II1, UNC-231 II1, and OP-4281II1 in the absence of the wild-type allele (Figures E1A, E1C, and E1D); this variant was identified in the heterozygous state in a sib-pair, UNC-186 II1 and UNC-186 II2 (Figure E1F). This sib-pair was also heterozygous for *WFDC2* variant c.307C>T, p.Cys103Arg; this variant was also identified in the homozygous state in individual CSU-150 II2 (Figure E1H). Because we identified *WFDC2* variant c.145T>C, p.Cys49Arg in five unrelated families, we Sanger sequenced *WFDC2* exon 2 in 1,229 additional individuals with chronic airway disease, revealing this variant in a heterozygous state in OP-398 II1 and a homozygous state in OP-1837 II1 (Figure E1B). Sanger sequencing of all four *WFDC2* coding exons in OP-398 II1 revealed another variant, c.271G>A, p.Gly91Ser, in addition to c.145T>C, p.Cys49Arg (Figure E1E). The missense variant c.271G>A, p.Gly91Ser also affects a highly conserved amino acid residue (Figure 1C). Furthermore, WES identified individual OP-4474 II1 to carry *WFDC2* variant c.326G>A, p. Cys109Tyr in the absence of the wild-type allele (Figure E1I).

Because of the high frequency of ultrarare variant c.145T>C, p.Cys49Arg in our cohort (12 of 22 alleles from 8 of 11 individuals), we examined whether it is a founder or a hot-spot variant. Of the 18 human WFDC family members, 14 are encoded by genes clustered within a narrow region on chromosome 20q13 (11, 12) (Figure 1A). Reanalysis of this *WFDC* gene cluster in WES/whole-genome sequencing data of OP-2147 II1 as well as OP-2032 II1 and his parents indicated the cosegregation of this variant with several SNPs, some of which are very rare (Figure E2). We also analyzed these SNPs in individuals OP-1837 II1 and OP-4281 II1 and his father, OP-4281 I1. In the 5′-direction of c.145T>C, the first non-cosegregating SNP is present at a distance of 26,425 bp (rs2745064); in the 3′-direction, SNP rs781204355 is present at a distance of 102,771 bp in the heterozygous state (Figure E2). Overall, 11 SNPs (including very rare ones) covering a region of 129.2 kb cosegregate uniformly with the variant c.145T>C, p.Cys49Arg, strongly supporting its classification as a founder variant.

### Clinical Phenotype

**Figure 2.**
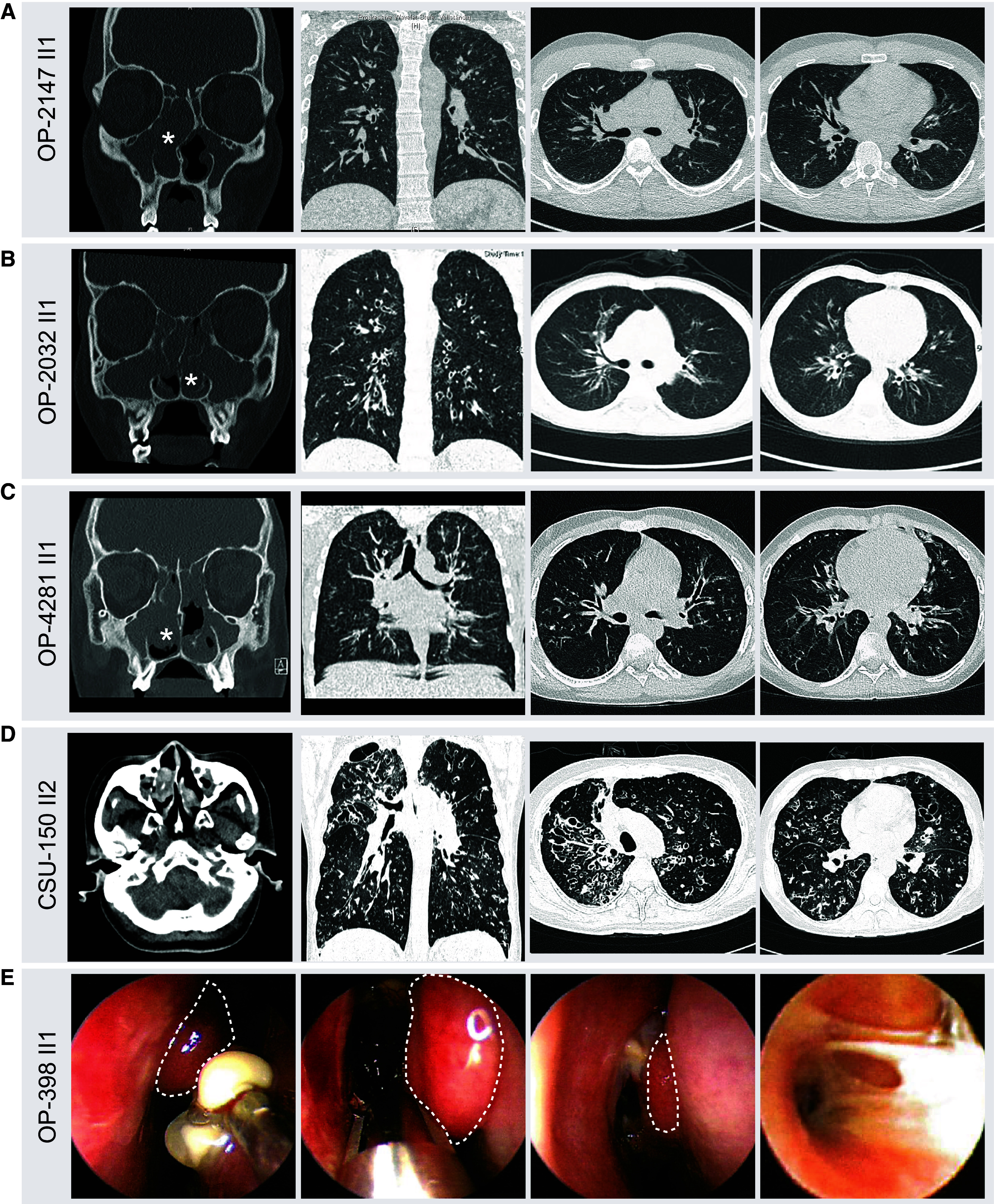
Individuals with biallelic *WFDC2* variants present with bronchiectasis in all lung fields as well as nasal polyposis. (*A–D*, left panels) Paranasal sinus computed tomography (CT) images of individuals OP-2147 II1, OP-2032 II, OP-4281 II1, and CSU-150 II2 show chronic rhinosinusitis with pronounced nasal polyposis (marked by asterisks). (*A–D*, middle and right panels) In addition, thoracic CT images of these individuals indicate bronchiectasis and bronchial wall thickening present in all lung fields. (*E*) Nasal endoscopy of OP-398 II1 demonstrates several nasal polyps (dashed lines) and plugging of thick mucus (right image).

All individuals with biallelic *WFDC2* variants presented with marked chronic respiratory symptoms affecting the upper and lower airways, and 9 of 11 individuals showed bronchiectasis by CT imaging (Figures 2A–2D and Table 1). Of note, the lungs of individuals with *WFDC2* mutations showed a remarkable involvement of the upper, middle, and lower lobes, resembling findings in CF, in which the upper lobes are typically the sites of greatest damage, which then expands to involve all lobes. This is distinct from genetic respiratory diseases such as PCD, where, classically, the middle and lower lung lobes are predominantly affected (13). Furthermore, nine individuals with *WFDC2* mutations suffered from chronic *P. aeruginosa* infection, which is known to be a risk factor associated with bronchiectasis (14).

Spirometry data were available in 9 of 11 individuals, and all demonstrated altered lung function parameters consistent with a chronic obstructive airway disease, resembling findings in CF and PCD. FEV_1_% predicted was reduced in all affected individuals examined (Table 1). Lung disease in subject UNC-186 II2 progressed to the point that lung transplantation was necessary.

In addition, all 11 individuals showed severe upper airway disease manifesting as CRS with pronounced nasal polyposis and progressive broadening of the nasal pyramid (Figure 2E and Table 1). Interestingly, very low nasal nitric oxide production rate was a shared finding in individuals with *WFDC2* mutations (9/9), which is associated with CRS (15) but also characteristic for PCD (16, 17) and a potential finding in CF (18). CF was specifically ruled out by the absence of disease-causing *CFTR* variants in all 11 individuals and by normal sweat chloride testing (9 of 11 individuals tested) (Table E2); furthermore, no disease-causing variants in reported PCD-causing genes were detected in the 11 *WFDC2*-deficient individuals.

WFDC2 was originally identified in epithelia of the epididymis duct and proposed to support sperm function (19). Thus, we examined whether WFDC2 deficiency could affect sperm function and male fertility. Interestingly, sperm count, motility, and morphology were all within the normal range in OP-2147 II1 (Table E3 and Videos E1–E4). In line with these findings, he fathered two children. Likewise, individual UNC-186 II1 fathered a son (confirmed by genetic testing). However, his affected sister (UNC-186 II2) was unable to conceive, despite attempted intrauterine insemination. In addition, female individuals UNC-231 II1 and CSU-150 II2 reported the inability to conceive (Table E2). These findings suggest that *WFDC2* deficiency impacts female rather than male fertility and warrant further investigation.

### *WFDC2* Expression Analysis

**Figure 3.**
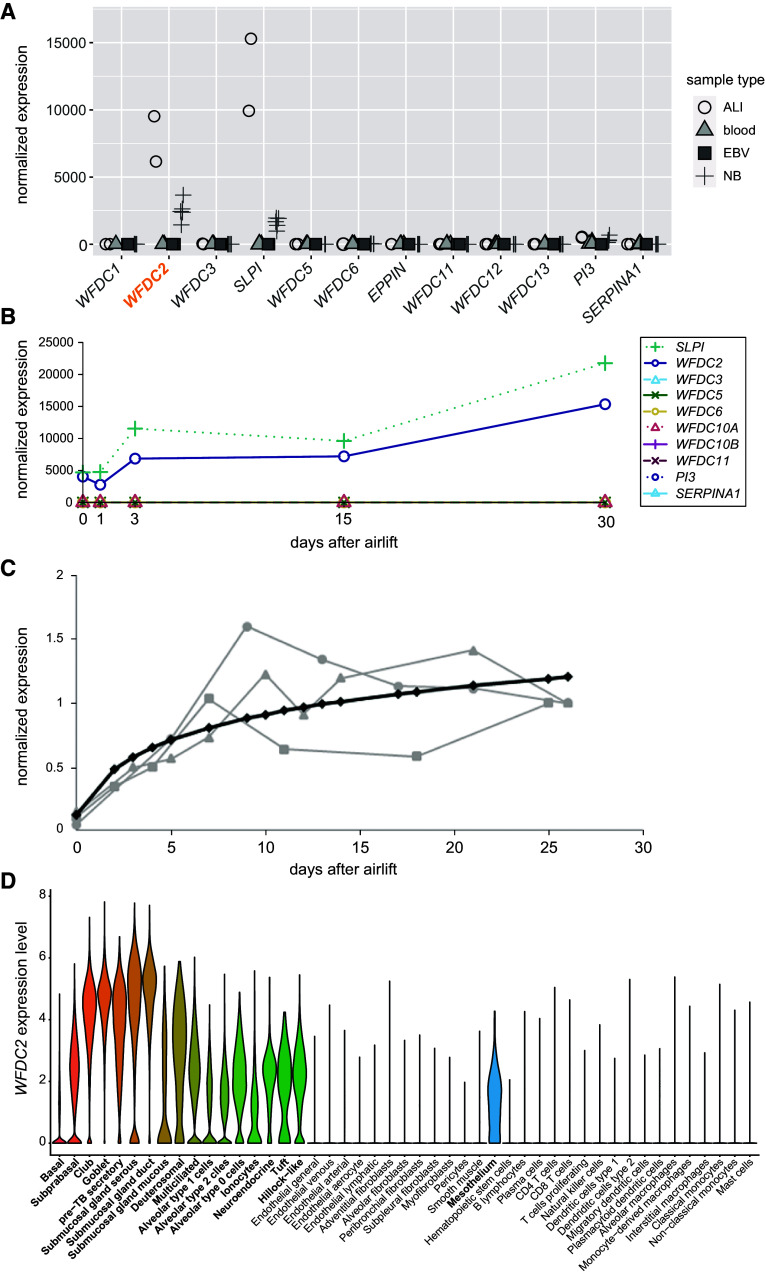
*WFDC2* and *SLPI* are the only WFDC domain family genes robustly expressed in respiratory epithelial cells. (*A*) Comparison of *WFDC* domain gene family expression by RNA sequencing from human nasal epithelial cells (NECs) obtained by NB, air–liquid interface cultured NECs (ALI), Epstein–Barr virus (EBV)-transformed lymphocytes (EBV), and whole blood (blood) demonstrates high expression of *WFDC2* and *SLPI* but no other *WFDC* family genes in respiratory cells. (*B*) Expression of *WFDC2* and *SLPI* follow a comparable pattern during ciliogenesis in ALI-cultured NECs (time points 0, 1, 3, 15, and 30 days after airlift). Other *WFDC* genes, including *WFDC1*, *EPPIN*, *WFDC8*, *WFDC9*, *WFDC12*, and *WFDC13*, are not detected. (*C*) *WFDC2* expression measured by quantitative PCR in bronchial epithelial cells cultured at the ALI from three healthy individuals (gray symbols; black line represents the data fitted to a mixed linear model). *WFDC2* is increasingly expressed in a differentiation-dependent manner. (*D*) Single-cell RNA sequencing of different nasal, airway, and parenchymal samples from 107 healthy human donors (23, 42) demonstrates that *WFDC2* is expressed primarily in secretory cells. Data shown here are the expression of WFDC2 per cell population in the merged dataset (biopsies, brushings, and dissections from all nasal, tracheal, bronchial, and parenchymal locations, from 107 healthy donors). NB = nasal brushing.

Other members of the WFDC protein family, such as SLPI (WFDC4) (20) and Elafin (WFDC14) (21), exhibit protease inhibitor activity and play an important role in the innate immune system. We examined the expression of all *WFDC* genes, including *WFDC2*, by RNA sequencing analyses (RNA seq) of *1*) native nasal epithelial cells (NECs) obtained by nasal brush biopsy (*n* = 5); *2*) air–liquid interface (ALI)-cultured NECs (*n* = 2); *3*) whole blood (*n* = 3); and *4*) epstein-barr-virus (EBV) transformed lymphocytes (*n* = 2) (Figure 3A). In addition, we investigated the expression of *WFDC* genes during differentiation in ALI-cultured NECs and bronchial epithelial cells (BECs) (Figures 3B and 3C). The results demonstrate low expression of all *WFDC* genes with the exception of *WFDC2* and *SLPI*. *WFDC2* expression increased throughout differentiation of NECs and BECs, and *SLPI* showed a comparable expression pattern during differentiation of NECs.

Furthermore, single-cell RNA seq of human samples from 107 healthy donors identified a unique expression pattern of *WFDC2* in secretory cells from tracheal, bronchial, and nasal samples, as well as in serous and ductal cells from submucosal glands (Figure 3D), which aligns with a separate dataset of control airway samples (Figure E3A). The canonical transcript (ENST00000372676) was the dominant one, as shown by RNA seq from nasal and bronchial samples (Figure E3B). These analyses provide evidence that *WFDC2* is expressed predominantly in secretory cells of the respiratory epithelium and submucosal glands.

**Figure 4.**
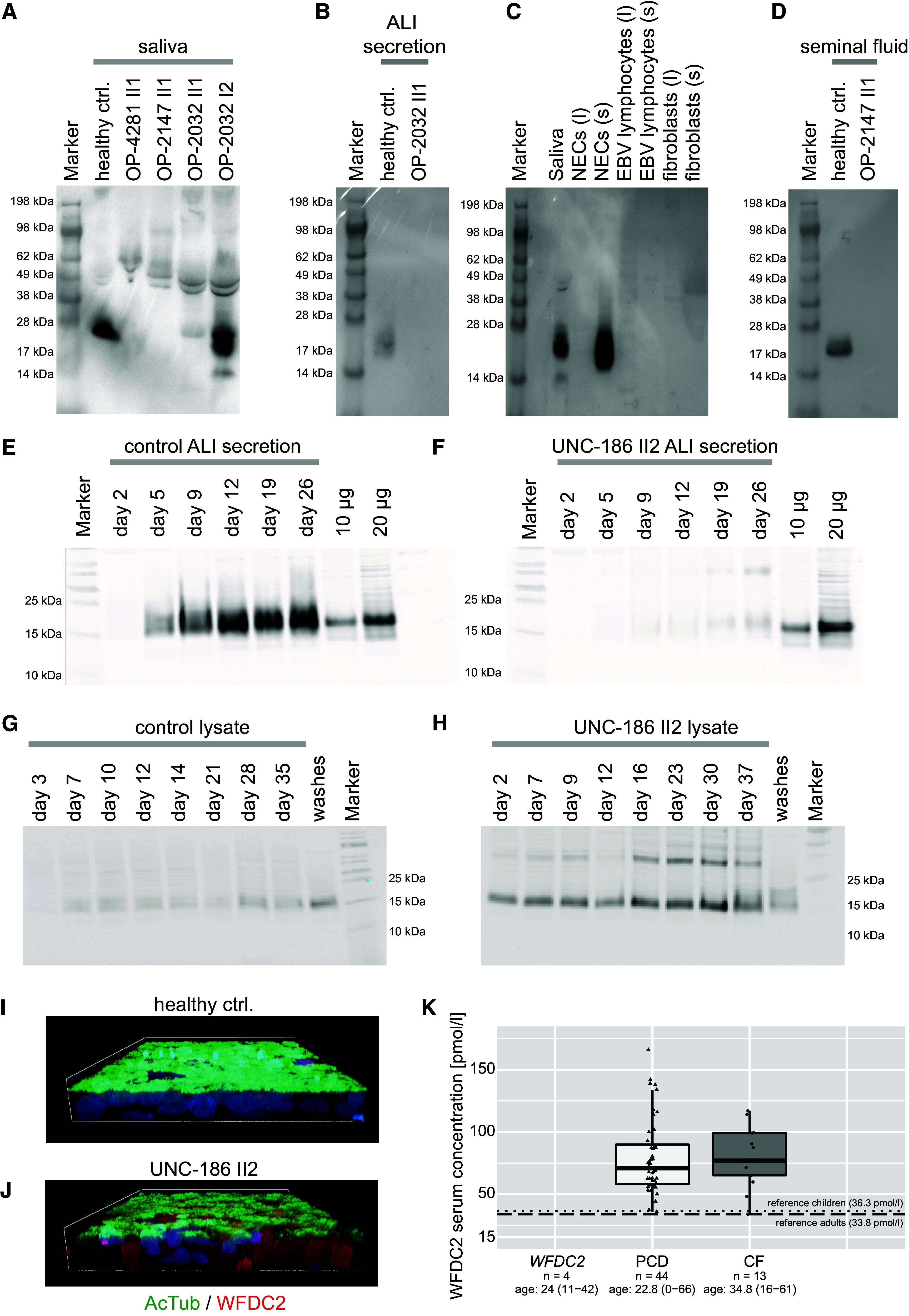
Secreted WFDC2 is detectable in healthy control but not *WFDC2*-deficient individuals. (*A*) Western blotting (WB) of saliva samples demonstrates the presence of WFDC2 (25 kD) in the healthy control and the healthy mother (OP-2032 I2) of individual OP-2032 II1. By contrast, this band is severely reduced or absent in saliva from individuals with pathogenic *WFDC2* variants (OP-4281 II1, OP-2147 II1, and OP-2032 II1). In healthy saliva, a diffuse protein band of approximately 25 kD that represents glycosylated WFDC2 is detectable. (*B*) WB reveals that WFDC2 is absent from apical secretions of air–liquid interface (ALI)-cultured nasal epithelial cells (NECs) of *WFDC2*-mutant individual OP-2032 II1. (*C*) By WB, WFDC2 is detectable in saliva and the apical secretions of ALI-cultured NECs of healthy individuals but not in NECs, fibroblasts, or EBV-transformed lymphocytes as well as supernatants of fibroblasts and EBV-transformed lymphocytes (l = lysate; s = supernatant). (*D*) WB demonstrates that WFDC2 is detectable in seminal fluid from healthy control but not *WFDC2*-mutant individual OP-2147 II1. (*E*) WB demonstrates that WFDC2 is detectable in apical secretions of healthy control bronchial epithelial cells (BECs) cultured at the ALI by Day 5, preceding ciliation, whereas (*F*) WFDC2 is weakly detectable in apical secretions from individual UNC-186 II2. A total of 10 and 20 μg of recombinant WFDC2 (HEK293 expression) is loaded as positive control. (*G*) In contrast, lysates from control BECs show low levels of WFDC2, whereas (*H*) UNC-186 II2 shows accumulation of WFDC2 reactive material. A total of 10 and 20 μg of recombinant WFDC2 (HEK293 expression) is loaded as positive control. (*I*) Whole mount immunofluorescence images of ALI-cultured BECs from a healthy control and (*J*) UNC-186 II2 stained with anti-WFDC2 (red), anti-acetylated tubulin to label cilia (green), and Hoechst 33342 to label nuclei. WFDC2 accumulates intracellularly in cells from UNC-186 II2. (*K*) Measurement of WFDC2 in blood serum shows that WFDC2 concentration is below the limit of detection in samples of individuals with biallelic *WFDC2* mutations UNC-186 II2, OP-2032 II1, OP-2147 II1, and OP-4281 II1 (OP-2032 II1 and OP-4281 II1 are tested twice, OP-2147 II1 is tested thrice with the same result). Median serum concentration of individuals with PCD (*n* = 44) and CF (*n* = 13) are elevated compared with published reference values (23) for healthy children (dotted line, 36.3 pmol/L, age 10–15 yr) and adults (dashed line, 33.8 pmol/L, age 23–38 yr). CF = cystic fibrosis; PCD = primary ciliary dyskinesia.

To investigate WFDC2 protein expression, we performed Western blotting (WB) of saliva, fibroblasts, EBV-transformed lymphocytes, and ALI-cultured NECs of healthy control subjects (Figures 4A–4C). WFDC2 was not detectable in any of the cell lysates or in cell medium from fibroblasts and EBV-transformed lymphocytes. However, a diffuse immunoreactive band of approximately 25 kD that represents the mature, glycosylated protein (22) was detected in saliva and apical secretions of healthy control ALI-cultured NECs. We next analyzed saliva, airway mucus (secreted in ALI-cultures of NECs), and seminal fluid from both healthy volunteers and individuals with *WFDC2* mutations by WB. In line with disease-causing *WFDC2* variants, the protein was not detectable in saliva of OP-2032 II1, OP-2147 II1, and OP-4281 II1, whereas in saliva of a healthy control individual and the heterozygous mother OP-2032 I2, the expected diffuse band at approximately 25 kD was detectable (Figure 4A). Comparing secretions of ALI-cultured NECs, we observed that WFDC2 is detectable in the healthy control individual but not individual OP-2032 II1 (Figure 4B). In addition, we demonstrated that WFDC2 was not detectable in seminal fluid in *WFDC2*-mutant individual OP-2147 II1 (Figure 4D), although this does not affect his ability to sire children.

In addition, we examined WFDC2 expression in ALI-cultured BECs of individual UNC-186 II2 during airway cell differentiation. WFDC2 is detectable in healthy control cultures at Day 5, which precedes ciliation (typically Day 11–13) and is continually secreted up to full mucociliary differentiation at Day 26 (Figure 4E). In contrast, WFDC2 was severely reduced in ALI secretions from UNC-186 II2 (Figure 4F). Interestingly, in cellular lysates from UNC-186 II2, we detected a strong WFDC2 signal that increased with differentiation of the culture, whereas the amount of intracellular WFDC2 in control cultures increased only slightly (Figures 4G and 4H). The abnormal accumulation of WFDC2 in respiratory cells from UNC-186 II2 was also detectable by immunofluorescence microscopy analysis (Figures 4I and 4J).

Using a commercial electrochemiluminescence immunoassay, we measured serum concentrations of WFDC2 in individuals with biallelic *WFDC2* variants and compared this to serum concentrations of WFDC2 in individuals with PCD and CF (Figure 4K). Among *WFDC2*-deficient individuals tested (*n* = 4), WFDC2 serum concentrations were below the detection limit. In contrast, individuals with PCD (*n* = 44) and CF (*n* = 13) presented with elevated WFDC2 serum concentrations, as previously reported (23).

### WFDC2 Structure Analysis

We performed homology modeling (24) to investigate the impact of *WFDC2* founder variant p.Cys49Arg on WFDC2 secretion. We found that both the *N*- and *C*-terminal regions of mature human WFDC2 had high sequence similarity to the sequence of the Nawaprin NMR structure (Protein Data Bank ID: 1 UDK). Our homology model resulting from this sequence similarity suggested that WFDC2 had two domains, each of which had a hairpin core surrounded by a loop that is tethered to the core via four disulfide bonds (Figure E4A), representing the signature motif of the whey acidic protein family (10). This is consistent with another independent human WFDC2 model (which was later released at the AlphaFold Protein Structure Database; Figure E4B), in that all 16 cysteines in WFDC2 form eight disulfides including the Cys49–Cys61.

**Figure 5.**
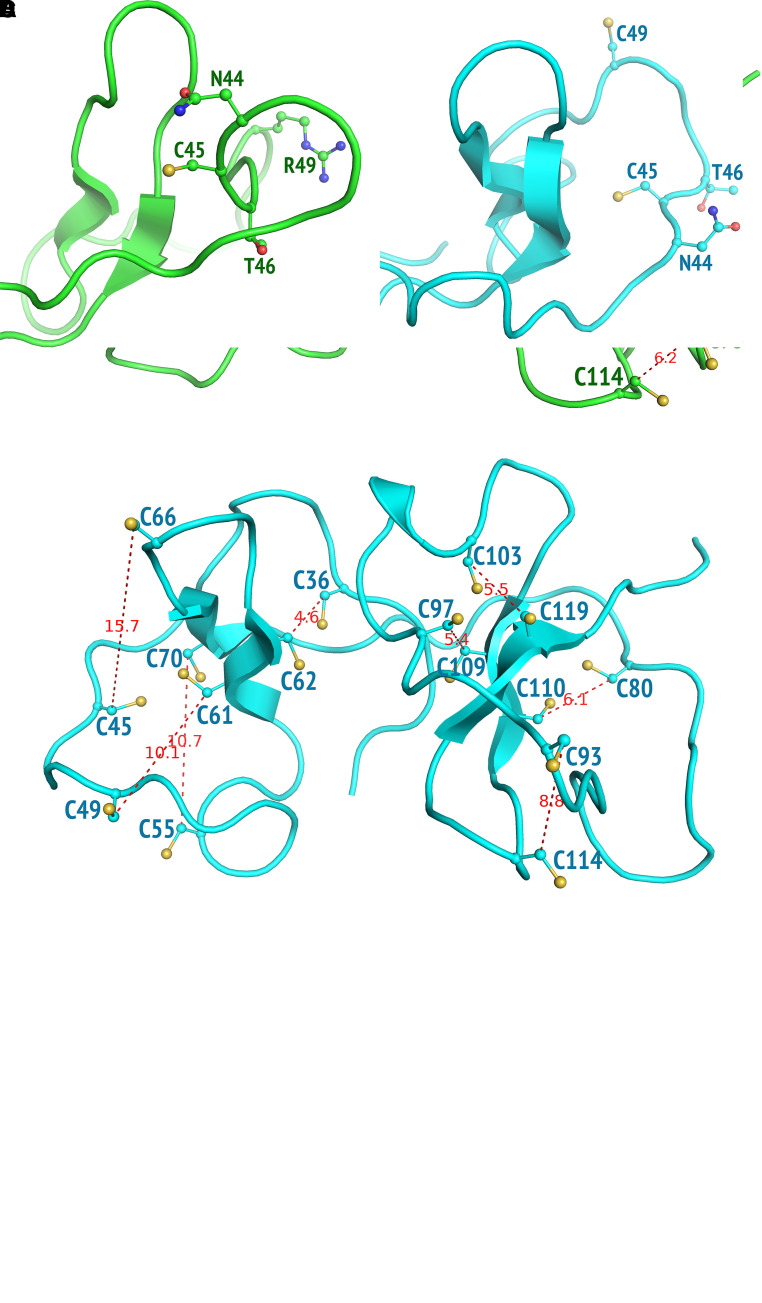
Disease-causing founder variant p.Cys49Arg impairs WFDC2 secretion through occlusion of the *N*-linked glycosylation site at Asn44. (*A*) The most populated conformation of the disulfide-free mature human WFDC2 with the Cys49Arg mutation showing the β carbon distances of seven disulfides (C36-C62, C45-C66, C55-C70, C80-C110, C93-C114, C97-C109, and C103-C119). (*B*) The most populated conformation of the wild-type disulfide-free mature human WFDC2 showing the β carbon distances of eight disulfides (C36-C62, C45-C66, C49-C61, C55-C70, C80-C110, C93-C114, C97-C109, and C103-C119). (*C*) Close-up view of the occluded glycosylation site of the Cys49Arg mutant. (*D*) Close-up view of the fully exposed glycosylation site of the wild type. Distances shown by dashed lines are in angstroms (Å). The most populated conformation of the mutant or wild type was derived from 220 distinct and independent simulations for each set, with an aggregated simulation time of 208.560 μs.

Furthermore, we performed two sets of physics-based molecular dynamics simulations of WFDC2 and its Cys49Arg variant with statistical relevance using forcefield FF12MC (25). For each set, 220 distinct and independent simulations were performed, with an aggregated simulation time of 208.560 microseconds (online supplement). In these simulations, each protein had all 16 cysteine residues in the reduced state representing the nascent WFDC2 without disulfides in the endoplasmic reticulum. This allowed us to probe the mutation effects on the conformation of Asn44Cys45Thr46, a known glycosylation site of WFDC2 (19) that is crucial to WFDC2 secretion (26). The most populated *C*-terminal domain conformations of the wild type and mutant were similar in that all eight cysteines were primed (positioned properly) to form four disulfides. However, the mutant *N*-terminal domain had four cysteines primed to form two disulfides (Figure 5A), whereas the wild-type *N*-terminal domain had only two cysteines primed to form one disulfide (Figure 5B). The latter underscores the necessity of *N*-linked glycosylation in the *N*-terminal domain to facilitate and ensure proper protein folding before secretion of WFDC2. Notably, Asn44 was partially occluded by the hydrophobic region of the hairpin core in the mutant (Figure 5C) but was fully exposed in the wild type (Figure 5D). This occlusion was primarily caused by the strong interresidue attractions of Asn44Cys45Thr46 to nearby residues in the *N*-terminal domain (Figures E4C and E4D) and suggested that the Cys49Arg variant disrupts *N*-linked glycosylation of the nascent protein in the endoplasmic reticulum and consequently impairs its secretion. This is consistent with the lack of detectable PNGase F-sensitive (hypoglycosylated) WFDC2 in saliva samples from individuals with *WFDC2* mutations (Figure E5).

### WFDC2 Functional Analysis

We next investigated ciliary integrity and mucociliary clearance capacity of *WFDC2*-deficient respiratory epithelium. High-speed video microscopy analysis of native NECs from OP-2032 II1, OP-2147 II1, and OP-4281 II1 indicated a normal, coordinated ciliary beat pattern; the ciliary beat frequency was reduced, but within the normal range (Videos E5–E12). Immunofluorescence microscopy analysis for ciliary components DNAH5, GAS8, and RSPH9 (*n* = 6) and transmission electron microscopy analyses (*n* = 8) in individuals with *WFDC2* mutations indicated normal ciliary ultrastructure (Table E2 and Figure E6).

To analyze mucociliary transport (MCT) in the absence of confounding inflammatory processes and other secondary effects, we cultured NECs from OP-2032 II1 and BECs from UNC-186 II2 at the ALI. High-speed video microscopy analysis demonstrated a normal ciliary beat frequency for OP-2032 II1 (5.7 ± 2.0 Hz; control, 4.9 ± 1.5 Hz at 25°C) and UNC 186 II2 (12.2 ± 0.2 Hz; control, 11.2 ± 0.4 Hz at 37°C) (Figure E7A and Videos E13 and E14). We also measured ciliary length and identified no difference between *WFDC2*-deficient cells of OP-2032 II1 and healthy control cells (Figure E7B). Cultures from both individuals with *WFDC2* mutations were capable of generating MCT in a directed fashion, with cells from UNC 186 II2 generating complete circular transport when cultured in an MCT device (27) (Video E15). Interestingly, the speed of MCT was low relative to the control cultures (Figures E7C and E7D), but the epithelial morphology and composition of cilia and centrosomes were comparable between healthy control subjects and OP-2032 II1 (Figure E8). Furthermore, particle-tracking microrheology analysis of mucus from ALI cultures of UNC-186 II2 and OP-2032 II1 indicated no difference in viscosity compared with control individuals (Figure E9). Taken together, our results suggest that *WFDC2*-deficient respiratory epithelia likely have a level of mucociliary clearance that is within the normal range.

### Proteomic Analysis

**Figure 6.**
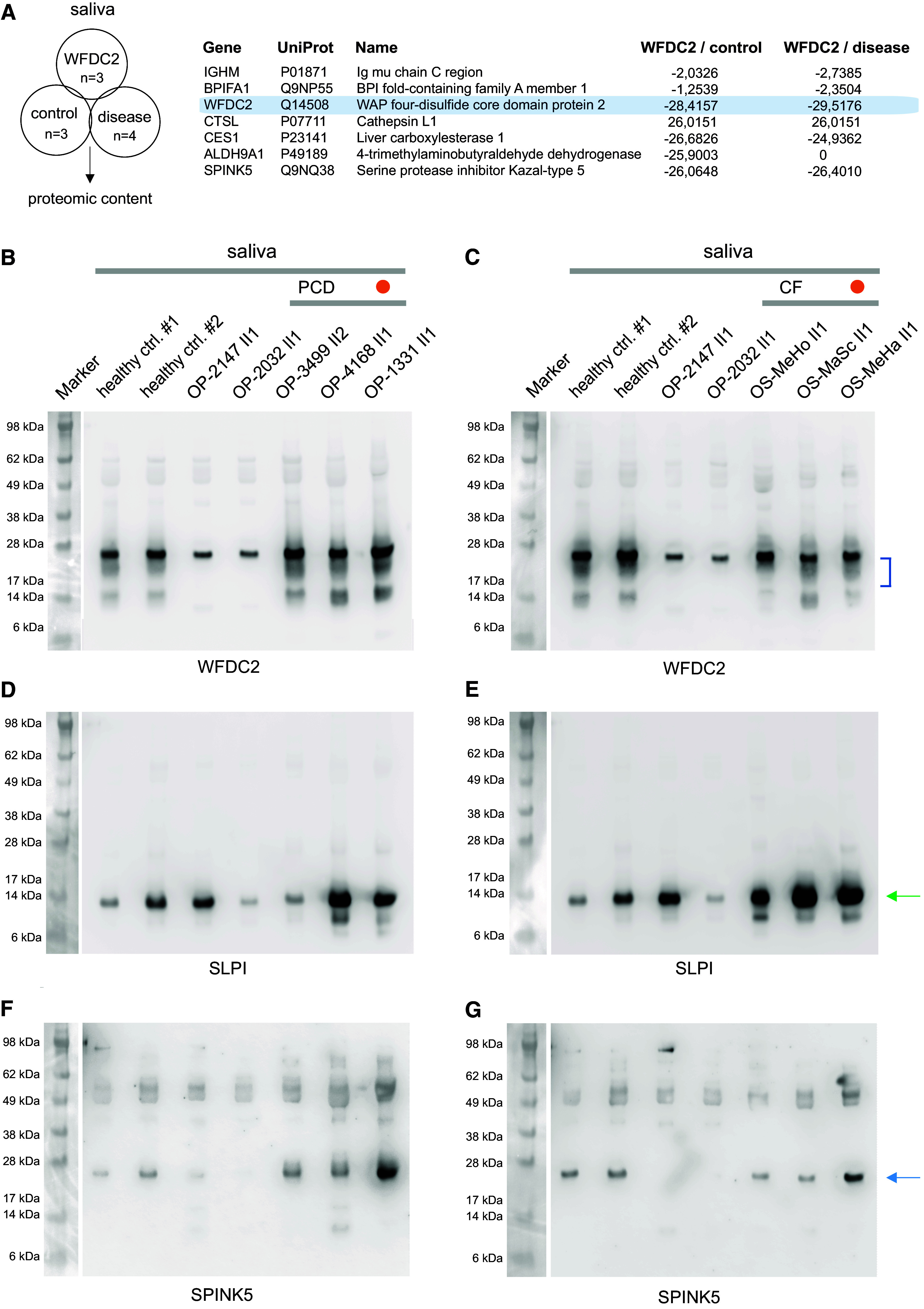
Proteomic content analysis of saliva among healthy control, *WFDC2*-mutant, and respiratory disease control groups. (*A*) Saliva from healthy control individuals (*n* = 3), individuals with *WFDC2* mutations (OP-2032 II1, OP-2147 II1, and OP-4281 II1), as well as control individuals with respiratory disease (primary ciliary dyskinesia [PCD], *n* = 2; cystic fibrosis [CF], *n* = 2) are subjected to liquid chromatography with tandem mass spectrometry (LC-MS/MS) to determine differentially expressed proteins. The gene symbol, UniProt identifier, and gene name, as well as log2 protein ratios of *WFDC2*-mutant compared with healthy control group (WFDC2/control) and *WFDC2*-mutant compared with respiratory disease control group (WFDC2/disease) are shown. WFDC2-specific peptides are not identified in the *WFDC2*-mutant group. The respiratory disease control group represents individuals with PCD OP-3499 II2 (*DNAH5*, c.10616G>A, p.Arg3539His + c.5557A>T, p.Lys1853*) and OP-3180 II1 (*SPAG1*, c.1282_1294del13, p.Ala428Profs*17, homozygous) as well as individuals with CF, OS-MeHo II1 and OS-MaSc II1. (*B* and *C*) WFDC2 Western blotting (WB) demonstrates a diffuse band of approximately 25 kD (blue brackets) that is detectable in healthy control subjects as well as individuals with PCD and CF but not individuals with *WFDC2* mutations OP-2147 II1 and OP-2032 II1. The immunoreactive band of approximately 26 kD in OP-2147 II1 and OP-2032 II1 is likely nonspecific, as this band does not shift in response to PNGase F (Figure E5), and LC-MS/MS does not identify WFDC2-specific peptides in individuals with *WFDC2* mutations. (*D* and *E*) SLPI WB demonstrates variable expression of an approximately 14-kD protein (green arrow) among healthy control subjects, individuals with *WFDC2* mutations, and control individuals with PCD and CF. (*F* and *G*) SPINK5 WB demonstrates significantly reduced expression of an approximately 26-kD protein (blue arrow) in individuals with *WFDC2* mutations compared with healthy and disease control groups. Patients with PCD are represented by OP-3499 II2, OP-4168 II1 (*RSPH1*, c.85G>T, p.Glu29Ter, homozygous), and OP-1331 II1 (*ODAD4*, c.425_426insT, p.Lys142AsnfsTer12, homozygous); patients with CF are represented by OS-MeHo II1, OS-MeSc II1, and OS-MeHa II1, which all harbor pathogenic *CFTR* variant c.1521_1523del, p.Phe508del in the homozygous state. Orange circle indicates individual with PCD, OP-1331 II1, and individual with CF, OS-MeHa II1, with bronchiectasis but no history of *Pseudomonas aeruginosa* infection. 10 μg of protein per sample is analyzed by WB.

To further understand the impact of WFDC2 deficiency, we analyzed saliva from healthy control individuals (*n* = 3), individuals with *WFDC2* mutations (*n* = 3), and control individuals with respiratory disease (PCD, *n* = 2; CF, *n* = 2) by liquid chromatography with tandem mass spectrometry (LC-MS/MS). Among more than 350 unique proteins identified in saliva, several proteins—including the serine protease inhibitor SPINK5—showed significantly altered expressions between *WFDC2*-mutant and healthy control groups as well as between *WFDC2*-mutant and respiratory disease control groups (Figures 6A and E10). We confirmed by WB that WFDC2 is robustly detectable in saliva from several individuals with PCD and CF, in contrast to individuals with *WFDC2* mutations (Figures 6B, 6C, E5C, and E5D). We analyzed SLPI by WB and, consistent with LC-MS/MS analysis, observed variable (not significantly altered) expression among healthy control, *WFDC2*-mutant, and respiratory disease control groups (Figures 6D and 6E). We also analyzed SPINK5 by WB and observed reduced expression of a proteolytically cleaved species (28) in individuals with *WFDC2* mutations compared with healthy control and respiratory disease control groups (Figures 6F and 6G).

## Discussion

Here, we describe a novel Mendelian disorder of chronic destructive airway disease characterized by bronchiectasis, chronic infection of the airways, pronounced CRS, and nasal polyposis due to autosomal recessive inheritance of pathogenic *WFDC2* variants. We show that WFDC2 is present in airway secretions, saliva, and seminal fluid in healthy control subjects but is hardly detectable, if at all, in these fluids and serum samples from affected individuals. This represents the first description of WFDC2 deficiency as a cause of chronic destructive airway disease, in contrast to PCD and CF, which show elevated expression of WFDC2. This agrees with previous reports in individuals with CF (23, 29) and recent studies reporting increased WFDC2 expression in interstitial lung disease (30–33). Bronchiectasis in *WFDC2* deficiency more closely mimics CF and IEI (all lobes) than PCD (predominantly middle and lower lobes). We recommend including WFDC2 deficiency in the differential diagnosis with CRS, CF, and PCD when nasal nitric oxide measurements are very low.

Our comprehensive genetic analyses show that the chromosomal region 5′ upstream of *WFDC2* as well as introns 1 and 2 contain highly repetitive DNA sequences due to many short interspersed nuclear elements. We detected two distinct heterozygous deletions in these regions spanning exons 1 and 2, probably due to recombinations of these elements. In addition, we identified the founder variant c.145T>C, p.Cys49Arg present in 12 of 22 alleles from 8 of 11 individuals. Protein structure analysis suggested that glycosylation at Asn44 is required for proper folding of the *N*-terminal domain of WFDC2. PNGase F analysis demonstrated that the hypoglycosylated WFDC2 protein was not detectable in saliva from three individuals harboring the Cys49Arg variant. This supports our conclusion that the Cys49Arg founder variant likely affects proper *N*-linked glycosylation and, consequently, its secretion.

WFDC2 has been reported to have antibacterial activity including against *P. aeruginosa* (22, 34, 35) as well as antiprotease activity (22, 34, 36). Proteomic analysis of saliva identified significantly altered expression of several proteins in individuals with *WFDC2* mutations, including the antimicrobial and antiinflammatory serine protease inhibitor SPINK5, whose reduced levels have been associated with CRS (37–39). This provides supporting evidence that WFDC2 serves a protective role in response to microbial infection and inflammation of the airways. Notably, we detect variable expression of SLPI in saliva among healthy control individuals, individuals with *WFDC2* mutations, and control individuals with disease (PCD and CF) by LC-MS/MS and WB. Nakajima and colleagues observed elevated *Slpi* expression in lung tissue from *Wfdc2*-null mice (40), whereas reduced SLPI expression has been reported in CF samples (29).

Mouse models deficient for WFDC2 exhibit respiratory failure and die in the neonatal period: defects include apoptosis of type-1 alveolar cells and thickening of the alveolar interstitium (41) and shortened cilia and impaired alveolar type-II function (40). We noted that ciliary length from affected individual OP-2032 II1 is not altered, and in ALI cultures from two individuals, the ciliary structure, beat frequency and coordination, as well as mucus viscosity were all within normal range. Although cytoplasmic accumulation of WFDC2 was noted in UNC-186 II2, obvious changes in cellular morphology or fate (also in OP-2032 II1) were not apparent. The phenotypic variation between human WFDC2 deficiency described here and mouse WFDC2 deficiency may reflect in part the sequence difference between human and mouse WFDC2, of which the latter contains a unique 52–amino acid linker region between WAP domains (Figure E11)

Although likely rare, *WFDC2* deficiency can now be screened for by genetic testing and established serum assays, and protein replacement therapy may be a potential treatment for this disease. Further studies on WFDC2 may provide insight into other causes of bronchiectasis and perhaps other pulmonary diseases.
